# A Case of Interstitial (Fallopian) Pregnancy

**Published:** 1850-01

**Authors:** Alexander McBride

**Affiliations:** Berea, Cuyahoga Co. Ohio


ART. IV.—*A case of Interstitial (Fallopian) pregnancy.* By ALEXANDER McBRIDE, M. D., of Berea, Cuyahoga Co. Ohio.

*October 18th.*—Mrs. G. aged 25 years, mother of two children, both living, was in good health, washing and cooking for several men; was seized, soon after 11 o'clock, with severe pain in the hypogastrium, which soon extended through the body, followed by vomiting, which was repeated often during the day, nothing of unusual appearance being ejected; pain continued very severe and she supposed it to be a "wind colic."

There being no female about the house but a young girl of not much experience, (her husband absent) it was not discovered that she was alarmingly ill till past 4, P. M. When I was called in it was 50 minutes past 4. I was struck with the pale and cadaverous appearance of the face; surface was cold and moist; pulse small, weak and frequent; (not enumerated by the watch) tongue pale and flabby; complained of pain in the hypogastrium, and between the shoulders, and throughout the body by turns, but constantly in the lower part of the abdomen and pelvis. At this time the pain in the hypogastrium, though constant, was not so severe as those darting through other parts of the body. The breathing was labored and sounded by the bed side as if a bronchus was obstructed with tough mucus. I enquired after the state of the lungs; she said she had a "bad cold," and if she had strength to cough she could clear her throat, which she did partially, soon afterwards.

I asked her, "Are you usually pale?" "No, I am usually very florid." "Are you pregnant?" "No." "Are your monthly turns regular?" "Yes, my last was about a month ago; it is now time for it again." (Her husband afterwards told me it had been five weeks.) She was not conscious of having been hurt in any way, or strained by heavy lifting, and knew no cause whatever of the present symptoms.

There was no tenderness of the abdomen. To relieve pain, and to stimulate, were the prominent indications. Gave a free dose of morph. acet. and ether sulph. In about fifteen minutes she vomited. Then gave morph. acet. $\frac{1}{4}$ gr. and calomel about 2 grs. She now was quiet, and somewhat easy for a half to three-quarters of an hour, but did not rally any; vomiting severely ensued. Gave again the same medicine. Soon after she had a desire to urinate, but could not void any. Said she had had great desire to urinate, but could not void any since 2 o'clock. The pain was now severe in the pelvis, and great distress through the body: countenance very anxious, losing heat rapidly. It now occurred to me that there might be a calculus impacted in the neck of the bladder; dispatched a messenger for my catheters (I was three miles from home), and addressed myself to the use of such means as might support the system till the pain could be relieved, This I attempted to do by opiates and stimulants internally, and heat and frictions externally. She said her "blood had stopped circulating," and "rub me," "I can't live long," and the like expressions. The agony was now intense, and she seemed conscious of approaching dissolution, throwing herself about, and making every exertion to sustain life and breathe. She threw herself out of bed into a chair, and called for warm water; a pail full was instantly had, and her feet put into it. I discovered now that her lower limbs were entirely cold. She was now sinking fast; her face was completely blanched; the pain was most intense in the hypogastrium, for there she held her hands, and faintly shrieked in agony. I lifted her into the bed, and continued the warm applications. She died about a quarter past 7 o'clock, two and a half hours after I was called in, and less than eight hours after the first appearance of the unpleasant symptoms.

Examination 37 hours after death; assisted by Dr. W. N. Longsworth; Dr. H. L. N. Leonard being present. Made an incision from the symphisis pubis towards the sternum, and from the same point into both iliac regions. The first morbid appearance that met the eye, was a large quantity of coagulum immediately within the peritoneum, occupying the pelvis and abdomen; this, with liquid blood, I removed to the amount of about three quarts. Among the coagulum in the pelvis, was found a well formed fœtus of about three months development (measured according to Devergie). On examining the uterus, I found about midway between the centre of the fundus amd the entrance of the right fallopian tube into the peritoneal side of the organ, a lacerated opening in which a finger's end might be placed, and a red spongy mass protruding; the surface for some distance around the opening was very vascular.

The uterus, with its appendages, was removed. In an hour afterwards the examination was further pursued. Dr. Hubbard being also present. The rupture was enlarged with the scalpel, when a cavity was laid open which contained a placenta, membranes, and a portion of the umbilicus. The surface of the cavity was similar to that of the gravid uterus. The cavity of the uterus was then laid open by carrying the scalpel from the os tincae towards the rupture, when it became evident that the first discovered cavity and that of the uterus were not continuous. The uterus was lined with a pulpy membranous substance, and within this was nearly an ounce of a light coffee colored mucilaginous fluid. A small probe was then passed from the fimbriated extremity of the right tube through its length into the anomalous cavity. The left tube was also passed by a probe, its passage terminating naturally in the uterus. An attempt was then made to find a passage from the strange cavity to the uterine cavity, without success; but on peeling the deciduous membrane from the surface of the right cornua, the canal of the tube was found, and a silver catheter, number six, was passed through it into the new cavity, distance about two lines.

The enigma was now solved, it was a case of development of the fœtus in that portion of the fallopian tube which is between the inner and peritoneal surfaces of the uterus. The peritoneal wall of the new-formed cavity offered the least resistance, and it ruptured, discharging the fœtus into the peritoneal cavity. The uterus is nearly twice the size of the non-gravid organ. In cutting into its substance to enlarge the rupture, large open vessels were observed. The surface of the os-uteri is granular.

The right ovarium is larger than the left, and exhibits near its center, a large corpus-luteum, having a cavity with a serous surface which would easily contain a large cranberry bean; the other ovarium appears in its normal state; both contain numerous graafian vessels.

I shall deposit the morbid specimen in the Cleveland Medical College, in good preservation, where it can be seen by the medical curious.

*Observations.*—1. Patient was nursing a child *nearly two years old.*

2. She had, as she supposed, colic three weeks before death.

3. She was *regular in menstruation* up to five weeks before the accident.

4. Death so soon followed the rupture, that peritoneal inflammation did not supervene.

5. Death was caused by the *loss of blood,* and the *pain* which was occasioned by its presence in the abdomen.

I have written the case at some length, because the cases which I have read have been briefly reported.

